# Peptide bonds revisited

**DOI:** 10.1107/S2052252525002106

**Published:** 2025-03-31

**Authors:** Santosh Panjikar, Manfred S. Weiss

**Affiliations:** a ANSTO Australian Synchrotron 800 Blackburn Road Clayton Victoria3168 Australia; b Department of Biochemistry and Molecular Biology Monash University Melbourne Victoria Australia; c Macromolecular Crystallography Helmholtz-Zentrum Berlin Albert-Einstein-Strasse 15 12489Berlin Germany; University of Michigan, USA

**Keywords:** peptide bonds, enol-like peptides, protonated carbonyl oxygen

## Abstract

Understanding the structural and chemical properties of peptide bonds within protein secondary structures is vital for elucidating their roles in protein folding, stability and function. This study examines the distinct characteristics of peptide bonds in α-helices and β-strands using a nonredundant data set comprising 1024 high-resolution protein crystal structures from the Protein Data Bank (PDB). The analysis reveals surprising and intriguing insights into bond lengths, angles, dihedral angles, electron-density distributions and hydrogen bonding within α-helices and β-strands. While the respective bond lengths (CN and CO) do not differ much between helices and strands, the bond angles (∠CNC_α_ and ∠OCN) are significantly larger in strands compared with helices. Furthermore, the peptide dihedral angle (ω) in helices clusters around 180° and follows a sharp Gaussian distribution with a standard deviation of 4.1°. In contrast, the distribution of dihedral angles in strands spans a much wider range, with a more flattened Gaussian peak around 180°. This distinct difference in the distribution of dihedral angles reflects the unique structural characteristics of helices and strands, highlighting their respective conformational preferences. Additionally, if the ratio of the electron-density values (2*mF*_o_ − *DF*_c_) at the midpoint of the CO bond and of the CN bond is calculated, a skewed distribution is observed, with the ratio being lower for helices than for strands. Moreover, higher normalized mean atomic displacement parameters (ADPs) for peptide atoms in helices relative to strands suggest increased flexibility or a more dynamic structure within helical regions. Analysis of hydrogen-bond distances between O and N atoms of the main chain reveals larger distances in helices compared with strands, indicative of distinct hydrogen-bonding patterns associated with different secondary structures. All of these observations taken together led us to conclude that peptide bonds in α-helices are different from peptide bonds in β-strands. Overall, α-helical peptide bonds seem to display a more enol-like character. This suggests that peptide oxygen atoms in helices are more likely to be protonated. These findings have several important implications for refining protein structures, particularly in regions susceptible to enol-like transitions or protonation. By recognizing the distinct bond-angle and bond-length variations associated with protonated carbonyl oxygen atoms, current refinement protocols can be adapted to apply more flexible restraints in these regions. This could improve the accuracy of modelling local geometries, where protonation or enol forms lead to subtle structural deviations from the canonical bond parameters typically enforced in refinement strategies.

## Abstract 2

High-resolution crystal structures reveal that peptide bonds in α-helices exhibit a slightly more pronounced enol-like character than those in β-strands. This can go as far as peptide oxygen atoms in protein structures being protonated.

## 1. Introduction

**Figure 1:**
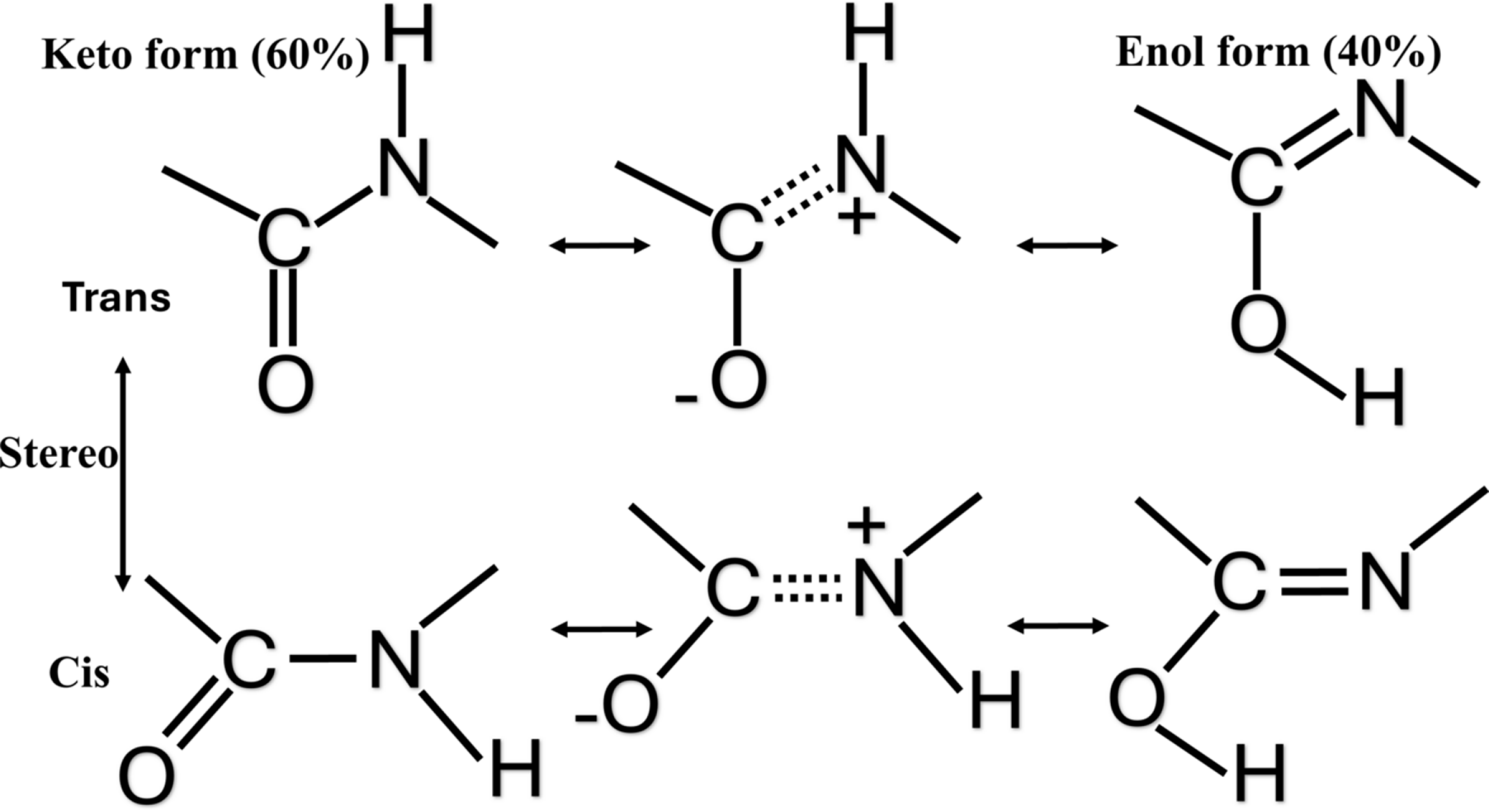
Schematic representation of the different tautomeric and isomeric states of the peptide bond. This figure illustrates the different tautomeric and isomeric states of the peptide bond, highlighting the interplay between keto–enol tautomerism and *cis*–*trans* isomerization. The first row represents the *trans* configuration, which is the predominant form in protein structures, while the second row depicts the *cis* configuration, a less common form that is sometimes observed in proline-containing peptides. The leftmost column shows the standard keto forms, where the carbonyl oxygen atom (C=O) retains its conventional bonding characteristics. The middle column illustrates resonance structures, demonstrating electron delocalization between the C=O and C—N bonds, which contributes to the partial double-bond character of the peptide bond. The rightmost column represents the enol forms, in which protonation of the carbonyl oxygen atom alters the electronic distribution, potentially affecting hydrogen-bonding interactions. The current understanding suggests that peptide bonds exist as a mixture between the keto and enol forms, with an estimated 60% keto character and 40% enol-like character. This schematic provides a conceptual framework for understanding how tautomerism and protonation influence peptide-bond chemistry, with implications for protein structure, stability and function.

Proteins are made out of amino acids, which are linked together by peptide bonds, The formation of a peptide bond involves a condensation reaction in which the amino group (–NH_2_) of one α-amino acid reacts with the carboxyl group (–COOH) of another. The resulting peptide bonds between carbon and nitrogen atoms exhibit diverse bond characters, including single, double or partial double-bond configurations. This is generally described as keto–enol tautomerism (Fig. 1 ▸). In proteins, it is generally believed that peptide bonds exhibit roughly 60% keto-like and 40% enol-like character (Isaev, 2015 ▸). This renders a peptide bond relatively rigid and leads to the arrangement that all participating atoms (C_α_, C, O, N_+1_, C_α+1_) lie in one plane. It also leads to the typically observed bond lengths, bond angles (Table 1 ▸) and electron-density distributions, with a partial negative charge being located on the peptide oxygen atoms and a partial positive charge being distributed over the NH group, with the hydrogen being more electropositive due to polarization.

Several studies have shown that significant distortions of the peptide bond from planarity are allowed in protein structures (Dunitz & Winkler, 1975 ▸; Ramachandran & Kolaskar, 1973 ▸; MacArthur & Thornton, 1996 ▸). More subtle deformations involve pyramidalization at either the carbonyl carbon or the nitrogen atom of the peptide bond (Improta *et al.*, 2015 ▸). Despite extensive experimental observations, the fundamental characteristics of the enol peptide group remain poorly understood. Density-functional theory (DFT)-based calculations have provided insight into the mechanisms underlying enol-to-keto tautomerism (Kamiya *et al.*, 2006 ▸). These studies demonstrate that the optimized structures of the enol-*trans* form in polyglycine with infinite length (PGI) have C=N and C—O bond distances of 1.269 and 1.392 Å, respectively, with an ∠COH of 109.5° and a carbonyl oxygen–hydrogen distance of 0.978 Å. In contrast, the keto-*trans* form exhibits C—N and C=O bond distances of 1.354 and 1.245 Å, respectively.

Even at high resolution, protein structures are typically refined with restraints, in which the peptide bond is treated as having a partial double-bond character. Since only one set of restraints is applied to all peptide bonds in a given structure, the C—N and C—O bond lengths in refined protein structures are clustered around the specific restrained values. In this study, over 1000 nonredundant crystal structures with a resolution better than 1.2 Å were analysed. The focus of the analysis was placed on bond lengths, bond angles, torsion angles, the midpoint electron density of the C=O and C—N bonds and the difference electron density near the carbonyl oxygen. The investigation was conducted to determine whether differences exist in the enol-like character of peptide groups across various secondary structures in proteins. Through the combination of high-resolution electron-density maps and computational analysis, findings were revealed that not only challenge current assumptions but also suggest new directions for protein structure refinement and functional interpretation.

## 2. Methods

### 2.1. Definition and assembly of the database

The study was conducted using data from the Brookhaven Protein Data Bank (PDB; Berman *et al.*, 2000 ▸; Bernstein *et al.*, 1977 ▸) as of April 2024. To ensure a nonredundant set of proteins, the *PISCES* protein sequence-culling server (https://dunbrack.fccc.edu/pisces) was utilized. Subsets of protein chains were culled based on criteria such as structure quality and maximum mutual sequence identity (Wang & Dunbrack, 2003 ▸). The selection criteria included (i) the inclusion of only X-ray structures with a resolution of 1.2 Å or better, (ii) a limitation of the maximum acceptable amino-acid identity between any two protein chains to 25% and (iii) setting the *R*-factor threshold to 0.2. All peptide atoms with alternate conformations were excluded, as such conformations may introduce variability in bond geometry that could obscure specific protonation effects. *Cis*-peptide bonds were also excluded, as they are less common and introduce significant steric strain, potentially affecting the consistency of the structural data.

Data sets with a resolution cutoff of 1.2 Å were selected to ensure that sufficient atomic-level detail would be available to resolve subtle features such as hydrogen atoms, bond angles and protonation effects. At this resolution, the electron-density maps usually provide high clarity, allowing the accurate interpretation of structural deviations and interactions. The chosen *R*-factor threshold of ≤0.2 ensures that the structural model aligns well with the experimental data, minimizing the risk of overfitting or inaccuracies in atom positions, which could affect the interpretation of protonation states.

Using these thresholds, 1135 PDB entries were identified. Further filtering based on the availability of processable electron-density maps (both 2*mF*_o_ − *DF*_c_ and *mF*_o_ − *DF*_c_) reduced this set to 1024 PDB entries comprising structures of polypeptides ranging from 40 to 1045 amino acids in length. The Wilson *B* factor for these proteins was found to range between 4.6 and 5.8 Å^2^, reflecting the overall thermal stability of the structures. Lower Wilson *B* factors were interpreted as indicative of more ordered structures, providing clearer electron density for interpreting subtle structural features such as protonation or enol-like behaviour at the carbonyl oxygen. By selecting data sets with lower *B* factors, the data were ensured to support accurate geometric and electronic analyses. The final set of proteins (Supplementary Table S1), referred to as the ‘25% database’, was used for all subsequent analyses. This name reflects the sequence-identity threshold applied during data-set curation, ensuring that no two protein chains share more than 25% sequence identity, thereby reducing redundancy and providing a more diverse representation of protein structures (Jabs *et al.*, 1999 ▸).

#### 2.1.1. Validation and robustness testing

To assess the robustness of the data set and minimize potential biases introduced by refinement parameters, additional filtering was performed based on stricter refinement criteria. Structures were further evaluated using more stringent *R*-factor and *R*_free_ thresholds, as well as refinement models incorporating anisotropic atomic displacement parameters (ADPs). A subset of structures refined at higher resolution limits was also examined to verify consistency with the main data set.

Additionally, previous analyses conducted using only structures with resolutions of 1.0 Å or better provided an independent validation of the observed geometric trends. Across different refinement criteria, the data set was systematically screened to ensure that findings were not artefacts of data selection but instead reflected intrinsic structural properties of peptide bonds.

### 2.2. Geometric calculations and secondary-structure assignment

In the initial processing step, a clean-up procedure was applied to all entries using *PROCHECK* (Laskowski *et al.*, 1993 ▸), ensuring consistency regarding nomenclature and other criteria. Subsequently, geometric parameters such as bond lengths, bond angles and dihedral angles were calculated from the coordinates. These calculations were performed using a custom Python program developed with the PDBParser library from BioPython (Cock *et al.*, 2009 ▸). A filtering approach based on *Z*-scores for bond angles and lengths was applied, and any peptide bonds falling beyond three standard deviations from the mean were removed.

The assignment of secondary structure was carried out using the *Definition of Secondary Structure of Protein* (*DSSP*) program (Kabsch & Sander, 1983 ▸; Touw *et al.*, 2015 ▸) available from the BioPython library. *DSSP* assigns seven different secondary structures, *i.e.* α-helix, 3_10_-helix, π-helix, extended β-strand, residue in isolated β-bridge, bend and hydrogen-bonded turn. Additionally, residues without any recognizable secondary structure are categorized as coil. Moreover, main-chain hydrogen bonds were identified and calculated using the *DSSP* algorithm.

### 2.3. Midpoint electron densities

Electron-density values at bond midpoints were extracted from 2*mF*_o_ − *DF*_c_ maps following standard refinement protocols. It is important to note that independent atom model (IAM)-based refinement does not explicitly account for electron redistribution across bonds. While the IAM remains the standard model for macromolecular refinement, it may underestimate electron density at bond midpoints due to its assumption of isolated, nonpolarized atoms. As such, these measurements should be considered as relative indicators of electronic variations between helices and strands rather than absolute values of bond order.

A custom Python program was developed to calculate the electron density at the midpoint of bonds (between C and O atoms and between C and N atoms) using the PDBParser library from BioPython and the densityAnalysis library from *pdb_eda*. The *pdb_eda* package (Yao & Moseley, 2020 ▸) is designed to provide classes and methods for analysing electron-density map data sourced from the Protein Data Bank (PDB).

The fromPDBid() function from the densityAnalysis module was utilized to create a densityAnalysis instance. This instance was constructed using only a PDB ID, facilitating seamless access to CCP4 data through the densityObj and diffDensityObj data members. These data members contain both header information and density maps from the CCP4 standard map file, with densityObj representing the 2*mF*_o_ − *DF*_c_ density map and diffDensityObj representing the *mF*_o_ − *DF*_c_ density map.

Various methods for analysing CCP4 data were made available through the densityAnalysis module. For example, electron density could be extracted from a given set of *xyz* coordinates, enabling efficient processing of electron-density maps for the analysis. The Python scripts used in this study are available from the authors upon request.

### 2.4. Difference density peak near carbonyl oxygen

To identify difference electron-density peaks near carbonyl oxygen atoms, the *CCP*4 program *MAPMASK* (Collaborative Computational Project, Number 4, 1994 ▸; Agirre *et al.*, 2023 ▸) was utilized in order to extract the electron density around the protein structure of interest. Following this, the *CCP*4 program *PEAKMAX* was employed to isolate difference density peaks exceeding 2σ, with a number of peaks selection threshold set to 5000. These identified peaks were saved in PDB format for detailed analysis in the subsequent steps.

Using the Bio.PDB module from the BioPython library, the protein structure was loaded and the PDB file containing the selected peaks was used to represent the peak atoms. For each residue in the protein, the carbonyl oxygen and carbon atoms were identified. The distance between each carbonyl oxygen atom and the nearby peaks was calculated, and peaks falling within the range 0.8–1.2 Å were classified as valid candidates.

For each valid peak, the angle ∠C–O–PEAK was computed to capture the spatial relationship between the C–O (carbonyl carbon-to-oxygen) vector and the O–PEAK vector. Peaks forming an angle between 80° and 120° were selected for further scrutiny.

Difference electron-density peaks were filtered based on both density characteristics and the peptide-bond geometry. Peaks were retained only if the electron-density ratio at the midpoint of the bond (C—O and C—N) was less than 1 and the dihedral angles of the peptide bond fell within a narrow range of 180 ± 5°. These difference density peaks near carbonyl oxygen atoms were initially identified through automated peak-detection and filtering methods. A representative subset of these peaks was then visually inspected using the *Coot* graphics software (Emsley & Cowtan, 2004 ▸; Casañal *et al.*, 2020 ▸) to confirm the validity of the automated approach and to ensure consistency in the protonation assignments.

### 2.5. Data visualization

To understand the underlying distribution of data, kernel density estimate (KDE) plots were utilized to visualize the distribution of various geometrical parameters discussed here. The probability density function (PDF) of a random variable is estimated using KDE plots. Unlike histograms, which divide the data into discrete bins, KDE plots provide a continuous estimate of the density. This is achieved by placing a ‘kernel’ (a smooth, symmetric function) at each data point, which are then summed to obtain the density estimate (Hastie *et al.*, 2009 ▸).

The plot is based on a kernel function; by default, the Gaussian kernel is used for each plot. The bandwidth (or smoothing parameter) controls the width of the kernel and, therefore, the smoothness of the KDE. A larger bandwidth results in a smoother density estimate, while a smaller bandwidth provides a more detailed estimate. The bandwidth used in each plot here is 1.

The vertical axis in these KDE plots is dimensionless, as it represents an estimated probability density rather than an absolute frequency count. This ensures that the density distributions are comparable across different data sets while maintaining relative proportions of occurrences.

For a set of data points *x_1_*, *x_2_*, *x_3_*, …, *x_n_*, the KDE at a point *x* is given by
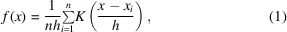
where *K* is the kernel function, *h* is the bandwidth and *n* is the number of data points. All of the KDE plots are produced using the Seaborn and Matplotlib libraries.

To complement the KDE plots, violin plots were also generated to provide a comparative statistical summary of bond geometry, hydrogen-bond distances and electron-density characteristics in helices and strands. Violin plots combine density estimation with statistical summaries, displaying the distribution spread alongside quartile markers. This dual representation ensures that both the overall distribution trends and the underlying statistical variations are effectively captured. While KDE plots (Fig. 3) highlight probability density distributions, violin plots (Supplementary Fig. S1) provide additional insight into the spread and variability of the data, strengthening the comparative analysis.

### 2.6. Structure refinement of PDB entry 6mu9

The β-lactamase structure (PDB entry 6mu9; Center for Structural Genomics of Infectious Diseases, unpublished work), originally deposited in the Protein Data Bank, was re-refined using *Phenix* (Liebschner *et al.*, 2019 ▸) in the resolution range 20–0.97 Å to investigate the impact of hydrogen placement and protonation on refinement quality and electron-density features. The deposited structure, refined by the original authors using *REFMAC*5 (Murshudov *et al.*, 1997 ▸), served as the starting point for all subsequent refinements. The *REFMAC*5 refinement excluded hydrogen atoms and reported *R* and *R*_free_ values of 10.1% and 11.1%, respectively.

Subsequent refinements included the following.

(i) Re-refinement without hydrogen atoms. The structure was re-refined in *Phenix* without hydrogen atoms, resulting in *R* and *R*_free_ values of 10.83% and 12.79%, respectively.
(ii) Re-refinement with hydrogen atoms in riding positions. Hydrogen atoms were added in riding positions, leading to *R* and *R*_free_ values of 9.39% and 11.05%, respectively.
(iii) Re-refinement with protonated carbonyl oxygen. A hydrogen atom was explicitly added to the carbonyl oxygen atom of residue 299 to represent a protonated state, and the hydrogen on the N atom of residue 300, connected to the peptide bond, was removed. Custom geometry restraints for the protonated carbonyl oxygen atom were generated using the *phenix.elbow* (Moriarty *et al.*, 2009 ▸) module to ensure proper bond lengths and angles. Hydrogen atoms for other atoms were retained in riding positions, while the hydrogen atom attached to the N atom of residue 299 was removed for validation purposes. This refinement resulted in *R* and *R*_free_ values of 9.40% and 11.05%, respectively.

All relevant refinement parameters for the models are given in Table 2 ▸.

For electron-density analysis, 2*mF*_o_ − *DF*_c_ and *mF*_o_ − *DF*_c_ maps from the *REFMAC*5 refinement were contoured at 1.8 and 0.35 e Å^−3^, respectively. Similarly, 2*mF*_o_ − *DF*_c_ and *mF*_o_ − *DF*_c_ maps from *Phenix* refinements were contoured at 2.8 and 0.37 e Å^−3^, respectively. Across all refinements, no difference density was observed at the N atom of residue 300.

All refinements were analysed using electron-density maps to assess structural accuracy. The protonated carbonyl oxygen atom at residue 299 was validated by the absence of residual difference density. Additional geometry validation was conducted using *Phenix* tools to ensure proper stereochemistry and atomic placement.

#### 2.6.1. Refinement analysis: effect of restraint relaxation on bond geometry

To evaluate whether observed deviations in bond lengths and angles are primarily a consequence of refinement restraints or are genuinely supported by the X-ray data, we performed additional re-refinement experiments under progressively relaxed geometric restraints using *Phenix*. For this analysis, the weight on geometric restraints (wc) was systematically reduced in refinements from wc = 0.5 (strong restraints) to wc = 0.0 (completely unrestrained). Additionally, the bond slack parameter was set to 0.02 Å, allowing greater variability in bond lengths and angles while still maintaining a physically reasonable structure.

The results showed that as restraints were progressively relaxed

(i) the bond-length r.m.s.d. increased from 0.007 Å (strict restraints) to 0.075 Å (fully relaxed restraints),
(ii) the bond-angle r.m.s.d. increased from 1.111° to 3.893° and
(ii) *R*_work_ and *R*_free_ remained relatively stable across all refinements, ranging from 9.94% to 10.24% and from 11.39% to 11.58%, respectively

These observations indicate that the observed deviations in peptide-bond geometries are at least partially supported by the X-ray data themselves rather than being entirely imposed by refinement restraints. The greater variation in bond angles compared with bond lengths suggests that secondary-structure-dependent differences in bond geometry reflect real structural flexibility, particularly in β-strands, which accommodate greater conformational adaptability. Thus, this additional refinement analysis confirms that the observed bond-length and angle distributions in helices and strands are not merely artefacts of refinement constraints but are likely to be inherent to the protein structures.

## 3. Results and discussion

### 3.1. The 25% data set

The nonredundant set of proteins in our 25% database contains a total of 1024 proteins with 215 230 peptide bonds. 64 061 peptide bonds belong to α-helices and 50 787 peptide bonds to extended β-strands; the remaining 100 382 peptide bonds belong to other secondary structures.

The amino-acid composition of this set agrees very well with that derived from sequence data (McCaldon & Argos, 1988 ▸). The correlation coefficient between the two sets of numbers is 0.97. This shows that the selected set of proteins is representative and that it therefore forms a solid basis for statistical analysis.

### 3.2. Geometrical parameters

#### 3.2.1. Bond lengths and bond angles

**Figure 2:**
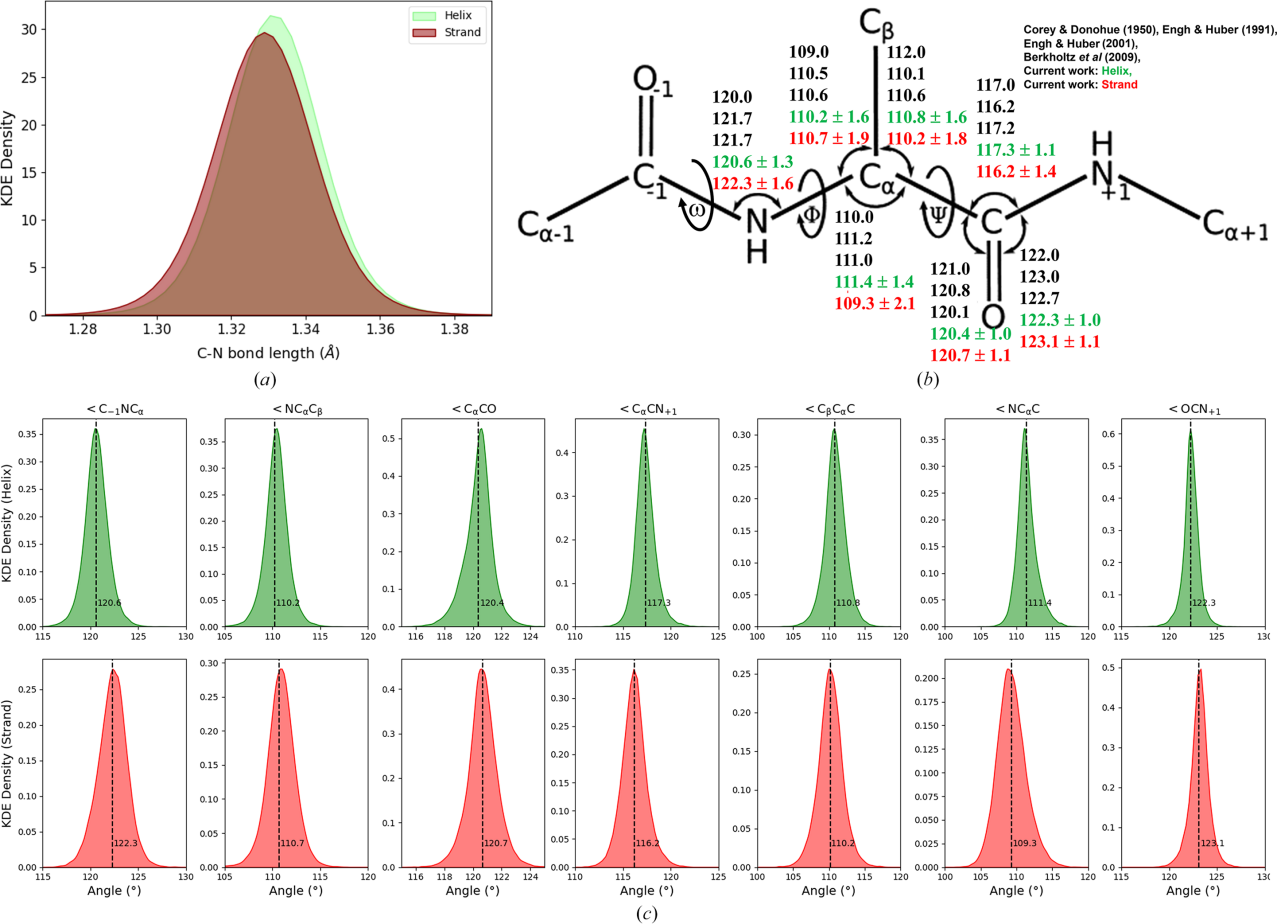
(*a*) KDE of C—N bond lengths in helix (light green) and strand (dark red) conformations. The density distributions demonstrate a slight difference in the peak positions of C—N bond lengths, with helices exhibiting a slightly higher peak density at approximately 1.32–1.33 Å compared with strands. The significant overlap in distributions indicates comparable bond lengths between the two secondary structures. (*b*) The backbone geometry of the central residue (residue 0) is depicted, including atoms from the adjacent residues (−1 and +1) that form its two peptide units. Seven bond angles associated with residue 0 are labelled, with ideal values listed from four key references in the descending order Corey & Donohue (1950 ▸), Engh & Huber (1991 ▸, 2001 ▸) and Berkholz *et al.* (2009 ▸). Modern refinement and modelling programs commonly use the 1991 or 2001 Engh and Huber values or slight variations thereof. Rotatable bonds defining the backbone torsion angles ω, φ and ψ are also shown. The figure, adapted from Berkholz *et al.* (2009 ▸), incorporates updates from the current work, with new data for helices highlighted in green and strands in red. Additionally, standard deviations for each bond angle in helices and strands have been included to provide a quantitative measure of structural variability in each secondary structure. (*c*) KDE plot of seven key bond angles associated with peptide geometry. The top row represents the distributions of bond angles in helices, while the bottom row corresponds to those in strands. Each plot is based on data from a nonredundant set of 1024 high-resolution protein structures. The distributions illustrate distinct conformational preferences, highlighting the characteristic differences between helices and strands. Each plot includes a vertical dashed line (axvline) indicating the mean value of the angle, providing a visual representation of central tendencies within helices and strands. These mean values are indicated in Fig. 2 ▸(*b*).

Minimal differences in bond lengths (C—N and C—O) were observed between helices and strands. Both secondary structures were found to occupy a small portion of the broader C—N bond range, which typically spans from 1.28 to 1.38 Å. The bond lengths in helices and strands were clustered around 1.331 Å (σ = 0.010 Å) for α-helices and 1.329 Å (σ = 0.010 Å) for β-strands, indicating minimal variation between the two secondary structures and also indicating that neither secondary structure exhibits the full range of flexibility possible for the C—N bond, as shown in Fig. 2 ▸(*a*). This restriction to a narrow range is unsurprising, as these values are often applied as target restraints during crystallographic refinement. Deviations from this range, such as values closer to 1.28 or 1.38 Å, are indicative of different bond characteristics, such as partial double-bond character (closer to 1.28 Å) or more single-bond character (closer to 1.38 Å). This suggests that the peptide bonds in both helices and strands share a partial double-bond character, which is a hallmark of peptide-bond stability in these structures.

In β-strands, bond angles such as ∠C_−1_NC_α_ and ∠OCN_+1_ tend to be slightly larger compared with α-helices, suggesting a less constrained geometry. The extended nature of β-strands allows greater flexibility in accommodating hydrogen bonds, whereas α-helices enforce stricter angular constraints due to their tightly packed structure and repetitive hydrogen-bonding pattern [Figs. 2 ▸(*b*) and 2 ▸(*c*)]. In contrast, α-helices maintain more constrained bond angles to preserve the compact and ordered hydrogen-bonding network along the helical axis. The regularity of these bond angles (∠C_−1_NC_α_ and ∠OCN_+1_) is essential for stabilizing the helical structure, where the carbonyl oxygen atom of each residue forms a hydrogen bond to the amide group of a residue four positions earlier in the sequence. Any deviation from these typical bond angles in helices would likely lead to local disruptions in the hydrogen-bonding network, potentially destabilizing the helix.

To evaluate whether these observed differences in bond lengths and angles arise from refinement constraints rather than genuine structural effects, we performed re-refinement of the β-lactamase structure PDB entry 6mu9 with progressively relaxed geometric restraints. This analysis revealed that while the bond-length deviations remained relatively small (r.m.s.d. of 0.007 → 0.075 Å), the bond-angle deviations increased significantly (r.m.s.d. of 1.111° → 3.893°). Notably, the *R*_work_ and *R*_free_ values remained stable across refinements, suggesting that the variability in bond angles, particularly in β-strands, reflects intrinsic conformational flexibility rather than refinement-imposed constraints. This finding supports the hypothesis that helices maintain stricter bond geometries, while β-strands accommodate greater angular adaptability, consistent with their distinct hydrogen-bonding patterns.

To assess whether deviations in peptide-bond lengths and angles are influenced by local atomic displacement parameters (ADPs), bond-geometry variations were normalized relative to mean backbone ADPs. A regression analysis between bond geometry and ADP values revealed no significant correlation (*p* > 0.05), and *Z*-score normalization confirmed that observed differences persisted independent of thermal motion effects. These results support the interpretation that peptide-bond geometries are modulated by secondary-structure context rather than ADP variability alone, reinforcing the intrinsic nature of these geometric deviations.

Our analysis of peptide-bond geometry in α-helices and β-strands is further supported by prior ultrahigh-resolution structural studies. Zarychta *et al.* (2015 ▸) performed a multipolar atom model-based analysis of cholesterol oxidase and provided precise measurements of bond lengths and angles in different secondary-structure elements. Their study demonstrated slight but measurable variations in peptide-bond lengths and backbone conformations, which correlate well with our findings. Specifically, the observed bond-angle differences between helices and strands in our data set are in agreement with their refined structural parameters, highlighting how secondary structure is able to modulate peptide-bond geometry.

#### 3.2.2. The dihedral angle (ω)

**Figure 3:**
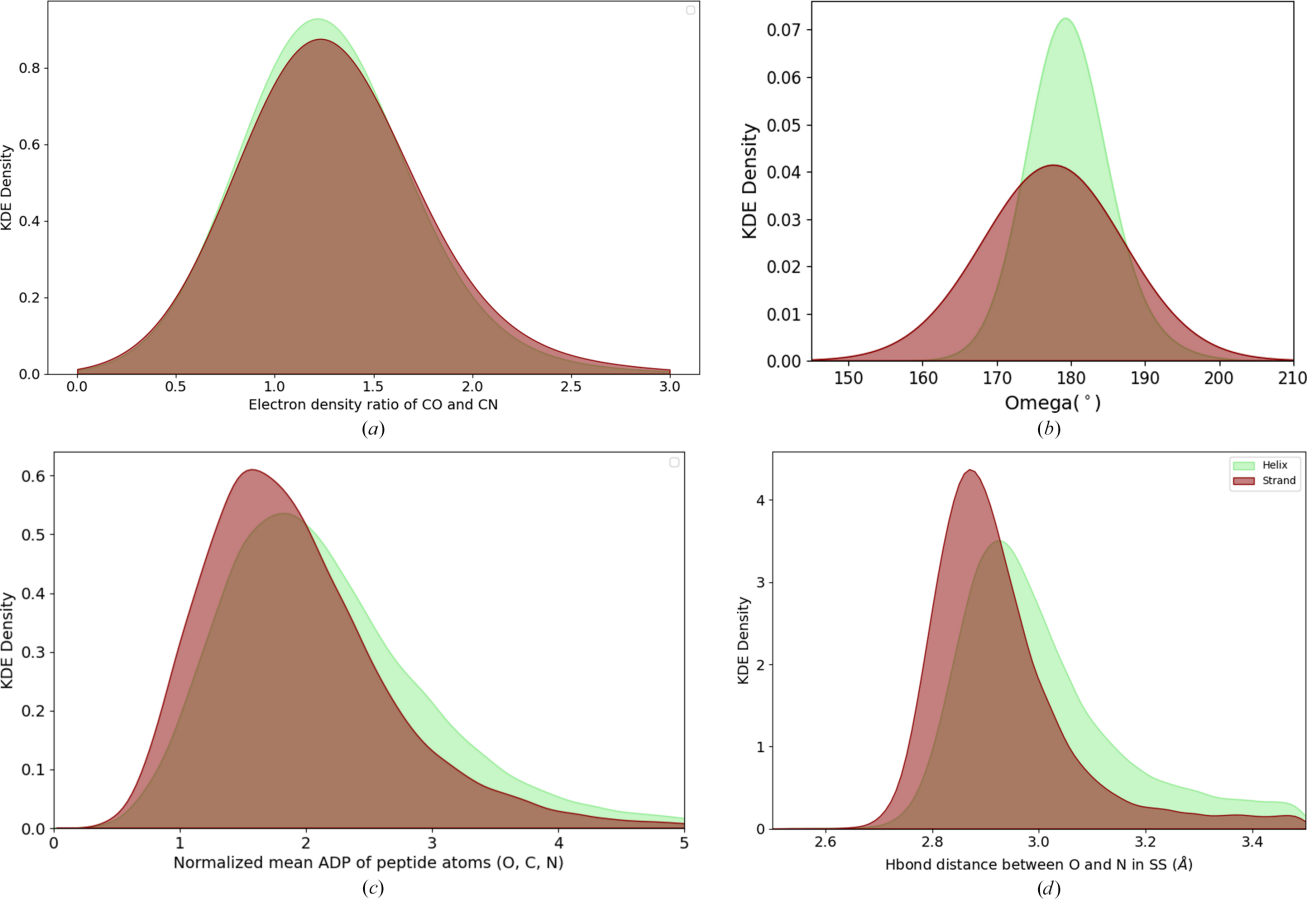
Comparative analysis of peptide geometry and properties in helices (light green) and strands (dark red). Data are derived from a nonredundant set of 1024 high-resolution protein structures. (*a*) KDE plot of *D*_ratio_, defined as the ratio of electron density at the midpoint of the carbonyl (C=O) bond to that of the amide (C—N) bond, comparing helices (light green) and strands (dark red). The density distributions reveal subtle differences in electron-density patterns, indicative of structural and electronic variations between the two secondary structures. (*b*) KDE plot of the distribution of the dihedral angle ω for helices (light green) and strands (dark red). The distributions illustrate the planarity of peptide bonds, with helices exhibiting a narrower distribution tightly centred around 180°, indicative of their structural rigidity. Strands display a broader distribution, reflecting increased geometric variability and flexibility in their peptide bonds. (*c*) KDE plot of the normalized mean atomic displacement parameters (ADP) of peptide atoms (O, C, N) in helices (light green) and strands (dark red). The distributions highlight the differences in atomic mobility between the two secondary-structure types. Helices display a broader range of ADP values, suggesting increased flexibility and dynamic movement compared with strands, which show a sharper peak indicative of greater rigidity. Higher ADP values typically correspond to greater atomic mobility or structural flexibility. (*d*) KDE plot of hydrogen-bond distances between main-chain nitrogen and oxygen atoms, comparing bonding patterns in helices (light green) and strands (dark red). The distributions illustrate distinct preferences in hydrogen-bond lengths, with helices showing a broader range and longer average bond distances, while strands exhibit shorter and more consistent distances. Hydrogen bonding serves as a key stabilizing force in both secondary structures.

The values for most of the dihedral angles ω in α-helices are distributed such that they form a rather sharp Gaussian centred around 180° with a standard deviation of 4.1° [Fig. 3 ▸(*b*)]. This distribution reflects the structural rigidity conferred by the cooperative hydrogen-bonding network in α-helices, particularly the regular *i* to *i* + 4 hydrogen-bonding pattern.

However, the hydrogen bonds in helices, while essential for maintaining stability, are weaker and longer compared with those in β-strands, as confirmed by our analysis of hydrogen-bond distances [see Section 3.5, Fig. 3 ▸(*d*)]. This finding is consistent with previous studies (Baker & Hubbard, 1984 ▸; Hubbard & Kamran Haider, 2010 ▸), which report that hydrogen bonds in β-strands tend to be shorter and more linear due to the extended, planar nature of the structure. Additionally, violin-plot representations [Supplementary Fig. S1(*d*)] further illustrate these differences in hydrogen-bond distances, highlighting the broader distribution in helices compared with β-strands. This weakening of hydrogen bonds in helices is likely to support a higher C=N character in the peptide bond. The reasoning behind this is that stronger hydrogen bonds, such as those in β-strands, favour the keto form (C=O), while the weaker hydrogen bonds in helices provide a more favourable environment for the enol form (C=N). This distinct geometry and hydrogen-bonding pattern reinforce the rigidity of α-helices, restricting ω variability and supporting the stable helical structure critical for protein function and stability (MacArthur & Thornton, 1996 ▸).

In contrast, β-strands exhibit a wider ω distribution, spanning 145–220°, with a flattened Gaussian peak around 180° and a standard deviation of 6.9°, as shown in Fig. 3 ▸(*a*). This is further reflected in the violin-plot representation [Supplementary Fig. S1(*b*)], which captures the broader variability in ω values for β-strands, reinforcing their structural adaptability. The more varied geometry in β-strands reflects their flexible hydrogen-bonding patterns, which accommodate local distortions, bulges or staggered ends. These irregularities provide β-strands with an adaptable structure, allowing more substantial ω variability that facilitates conformational adjustments during folding or in response to mechanical stress, such as in fibrous or flexible regions of a protein. Thus, the distinct ω distributions illustrate how the regular hydrogen bonding in helices promotes structural rigidity, while β-strands provide mechanical resilience and adaptability, contributing to protein-folding dynamics and functional versatility.

When ω approaches 180° in helices, the peptide bond stabilizes the planar conformation, reinforcing *sp*^2^ hybridization and favouring the regular *i* to *i* + 4 hydrogen bonding. However, protonation of the carbonyl oxygen atom can disrupt this stability. Protonation of the carbonyl oxygen introduces a positive charge, altering its hydrogen-bonding capabilities and shifting the electronic distribution within the peptide bond. This leads to a decrease in the double-bond character of the C=O bond while simultaneously increasing the partial double-bond character of the C—N bond (C=N-like), consistent with the enol-like resonance form. This change can weaken or distort the *i* to *i* + 4 hydrogen bond locally, introducing subtle kinks or bends in the helix. In β-strands, where bond angles already indicate a more relaxed geometry, protonation may lead to increased flexibility in hydrogen bonding without significant disruption to the backbone. These distinctions highlight how the electronic environment of the peptide bond influences stability and flexibility in secondary structures, with implications for protein folding and function.

This issue is reminiscent of the ‘chicken-and-egg’ problem: which comes first, more C=N bonds in helices, and as a result, more protonated O atoms, or vice versa? It is difficult to discuss causality in this context, but it is clear that protonation and the structural preferences of the peptide bonds interact in a complex manner, affecting stability and flexibility in different secondary-structure elements.

### 3.3. Electron density at the midpoint of the bond

To assess the extent of double-bond character, the electron density in the 2*mF*_o_ − *DF*_c_ map at the midpoint of the C=O bond and the midpoint of the C—N bond in each peptide bond within the protein structures was calculated without interpolation. The electron-density values for the 64 061 peptides in helices range from 0.3 to 5.9 e Å^−3^, whereas for the 50 787 peptides in strands the values range from 0.3 to 5.8 e Å^−3^.

To quantify the differences in bond character, the *D*_ratio_ was calculated as the ratio of the electron density at the midpoint of the C=O bond to that of the C—N bond. A higher *D*_ratio_ was interpreted as indicating that the C=O bond possesses more double-bond character, while a lower value was taken to suggest that the C—N bond exhibits more double-bond character. For the KDE plot [Fig. 3 ▸(*a*)], *D*_ratio_ was clipped between 0.5 and 3.0. The mean value of *D*_ratio_ for α-helices is 1.27 with a standard deviation of 0.32, whereas the mean value for β-strands is 1.29 with a standard deviation of 0.34.

While electron density at bond midpoints provides a useful metric for assessing variations in bond character, these values are influenced by the refinement model employed. IAM-based refinements assume spherically symmetric atomic electron densities, which may underestimate electron density at bond midpoints due to the lack of explicit charge redistribution.

However, our refinement analysis of PDB entry 6mu9 using increasingly relaxed geometric restraints suggests that these variations in electron density align with real structural deviations rather than being an artefact of restraint bias. The stability of *R*_work_ and *R*_free_ across refinements further supports the reliability of these trends, indicating that the electron-density differences at C=O and C—N bond midpoints reflect genuine structural features rather than refinement-induced distortions.

To account for potential limitations of the IAM, our results were compared with previous high-resolution electron-density studies of peptide bonds. Lario & Vrielink (2003 ▸) analysed atomic resolution density maps of cholesterol oxidase and reported secondary structure-dependent differences in electron density, particularly in C=O and C—N bonds. Their observations align with our findings, where helices exhibit slightly lower electron-density ratios at the carbonyl bond midpoint compared with β-strands, suggesting subtle electronic variations. These differences likely arise due to distinct hydrogen-bonding environments in each secondary structure, further supporting our interpretation of electronic delocalization effects in peptide bonds. Additionally, Zarychta *et al.* (2015 ▸) performed multipolar refinement of ultrahigh-resolution structures, confirming variations in peptide-bond geometries and electronic distributions, further validating our observations.

The KDE plot indicates that most C—N or C=O bonds retain a partially double-bonded character in both secondary structures. This observation aligns with expectations, as standard refinement protocols impose restraints to enforce partial double-bond character. However, the data also suggest that in specific structural contexts either the C—N or C=O bond can exhibit a more pronounced double-bond character, highlighting the potential influence of secondary structure on bond delocalization.

### 3.4. Normalized mean atomic displacement parameters for peptide atoms

The mean atomic displacement parameters (ADP) for peptide atoms (C, N and O) were calculated from the 64 061 peptide bonds in α-helices and the 50 787 peptide bonds in β-strands. These values were normalized using the Wilson *B* factor of their corresponding PDB entries, referred to here as the ‘normalized mean ADP values’.

The normalized mean ADP values for peptide atoms are higher in α-helices compared with β-strands. The KDE plot [Fig. 3 ▸(*c*)] of the normalized mean ADP value spans from 0.25 to 7, showing peaks at 1.9 for β-strands and 2.2 for α-helices. To complement the KDE visualization, violin plots were generated [Supplementary Fig. S1(*c*)] to provide a detailed distribution of the normalized mean ADP values across helices and strands. The violin plot highlights the spread and median values, offering additional insights into the structural variability in peptide-bond flexibility.

Higher *B* factors typically indicate greater atomic mobility or flexibility within a protein structure. While helices are generally more compact and tightly packed than strands, the higher average *B* factor for helices suggests that atoms in these regions may exhibit greater structural dynamics or local movement compared with those in strands. This increased flexibility could be attributed to several factors, including differences in packing, intermolecular interactions or the local environment within the protein. By incorporating violin plots, we further emphasize the extent of variation in atomic mobility within different secondary-structure elements, reinforcing the statistical robustness of our observations.

### 3.5. Hydrogen-bond distance between O and N atoms of the main chain

Hydrogen-bond distances for the main chain in protein structures were calculated by identifying potential donor–acceptor pairs of atoms. This calculation was performed by measuring the distance between backbone nitrogen and oxygen atoms and determining the secondary structure of the residues involved in the identified hydrogen bonds. For main-chain hydrogen bonds, the donor atoms are typically the backbone nitrogen atoms, while the acceptor atoms are the backbone oxygen atoms from nearby residues. The analysis was conducted using 1024 nonredundant, high-resolution protein structures.

The KDE plot [Fig. 3 ▸(*d*)] of hydrogen-bond distances shows that the main-chain hydrogen bonds span between 2.6 and 3.5 Å, with a peak at 2.93 Å for β-strands and 3.00 Å for α-helices. To complement the KDE visualization, violin plots were generated [Supplementary Fig. S1(*d*)] to provide a detailed representation of the distribution of hydrogen-bond distances in helices and strands. Violin plots effectively capture both the density distribution and summary statistics, offering additional insights into the spread and variability of hydrogen-bond distances within each secondary structure. The violin plot emphasizes that while β-strands have a tighter, more constrained distribution, helices exhibit a broader range, suggesting increased local flexibility.

In fact, the shorter hydrogen-bond distances in β-strands suggest that hydrogen bonds in β-strands are stronger than those in helices, as shorter bonds typically correlate with stronger interactions. This observation aligns with previous studies, such as Baker & Hubbard (1984 ▸) and Hubbard & Kamran Haider (2010 ▸), which reported that hydrogen bonds in β-strands tend to be more linear and shorter due to the extended, planar nature of the structure. Conversely, in α-helices, the typical *i* to *i* + 4 hydrogen-bonding pattern results in slightly longer bond distances, as the helical structure introduces a small angular constraint to each bond, making them weaker in comparison.

To further validate these findings, the refinement analysis of PDB entry 6mu9 was performed using progressively relaxed geometric restraints. The results showed that while bond lengths remained relatively stable, bond angles displayed increased flexibility, particularly in β-strands. *R*_work_ and *R*_free_ values remained stable across all refinements, indicating that the shorter and more linear hydrogen bonds observed in β-strands are not solely a consequence of refinement constraints but likely reflect an inherent feature of β-sheet architecture.

While the majority of hydrogen bonds observed in this analysis follow the conventional single donor–acceptor interaction, the possibility of bifurcated hydrogen bonds must also be considered. In these cases, a single donor (N—H) or acceptor (C=O) may interact with two hydrogen-bond partners, leading to non-ideal bonding geometries. Bifurcated hydrogen bonds are more likely to occur in loop regions, β-turns and flexible sites of proteins where local structural distortions allow noncanonical interactions. Although these interactions are generally weaker than standard linear hydrogen bonds, they could contribute to local stabilization or dynamic adaptability of protein structures, particularly in regions undergoing conformational changes. Additionally, the occurrence of protonation at carbonyl oxygen atoms may influence the formation of bifurcated hydrogen bonds, as altered electronic distributions could modify hydrogen-bond acceptor properties. While this study primarily focused on conventional main-chain hydrogen bonds, future work incorporating angular analyses and explicit hydrogen positions could provide deeper insights into the role of bifurcated hydrogen bonding in secondary-structure stability.

While our analysis provides valuable insights into hydrogen-bond distances in secondary structures, certain assumptions and limitations must be acknowledged. (i) The analysis relies on O⋯N distances to infer hydrogen bonds but does not explicitly account for hydrogen atom positions. Key geometric factors such as O—H⋯N angles and O⋯H distances, which determine bond strength and linearity, are not included for the reason that the hydrogen positions are typically not determined experimentally. This could lead to overinterpretation in some cases. (ii) Variations in bond geometry within β-strands and α-helices, influenced by factors such as steric hindrance or local interactions, were not explicitly analysed. These factors could introduce subtle deviations from the reported trends. (iii) Despite using high-resolution structures, potential inaccuracies in disordered regions or less-ordered backbone conformations could affect the precision of the reported distances. (iv) Incorporating explicitly modelled or calculated hydrogen atom positions and including angular metrics (for example O—H⋯N angles) would enhance the reliability and depth of this analysis.

By addressing these limitations, future studies could further clarify the role of hydrogen bonds in secondary-structure stability and refinement model accuracy.

### 3.6. Protonated carbonyl oxygen atoms

A total of 1589 peptide bonds were identified with a difference density peak near the carbonyl oxygen atom, where the electron-density ratio at the midpoints of the bonds (C—O and C—N) was less than 1. The dihedral angles (ω) of these peptide bonds fell within a narrow range of 180 ± 5°, suggesting protonated carbonyl oxygen atoms. The contour levels of the difference electron-density map ranged from 2σ to 8σ. Among the identified protonated peptide bonds, 33.4% were located within helices and 20.3% in strands, with the remainder distributed across other secondary structures: 1.0% in residues forming isolated β-bridges, 4.0% in 3_10_-helices, 2.6% in π-helices, 15.2% in hydrogen-bonded turns, 8.9% in bends and the rest in coils.

The Ramachandran plot of residues with protonated carbonyl oxygens was examined. The interquartile range (IQR) of the φ and ψ angles, representing the 25th to 75th percentile of the data, was calculated to capture the majority of values. For helices, the IQR for φ was found to range from −67° to −61° and that for ψ from −44° to −33°. Similarly, for strands, the IQR for φ ranged from −126° to −91° and that for ψ from 119° to 140°. These findings suggest that the protonation of the carbonyl oxygen atom does not significantly disturb the typical backbone conformation of helices and strands. Residues with protonated carbonyl oxygens were observed to fall within the expected conformational ranges for these secondary structures. The fact that the angles remain within their respective IQRs indicates that protonation does not drastically affect the overall structural integrity of the secondary elements of the protein, at least in terms of backbone angles. However, other factors, such as side-chain interactions, stability, hydrogen-bond networks or local flexibility, may still be influenced by protonation.

When the carbonyl oxygen atom is protonated, the neighbouring nitrogen (N_+1_) atom is less likely to form a hydrogen bond, as the hydrogen-bond acceptor ability of the protonated oxygen is reduced. Protonation or enolization at the carbonyl oxygen atom influences the electronic distribution across the peptide bond in both strands and helices. In helices, such modifications could slightly alter the hydrogen-bonding pattern, but due to the rigidity of the helix the effects may be more localized, potentially causing minor distortions rather than widespread structural changes. However, in strands, where bond angles such as ∠C_−1_NC_α_ and ∠OCN_+1_ suggest a more relaxed geometry, as shown in Figs. 2 ▸(*b*) and 2 ▸(*c*), the effects of protonation could have a more pronounced impact on hydrogen-bonding interactions. Protonation in strand regions might lead to increased flexibility in the hydrogen-bonding network, without significantly disrupting the backbone, which is already more adaptable due to the larger bond angles.

**Figure 4:**
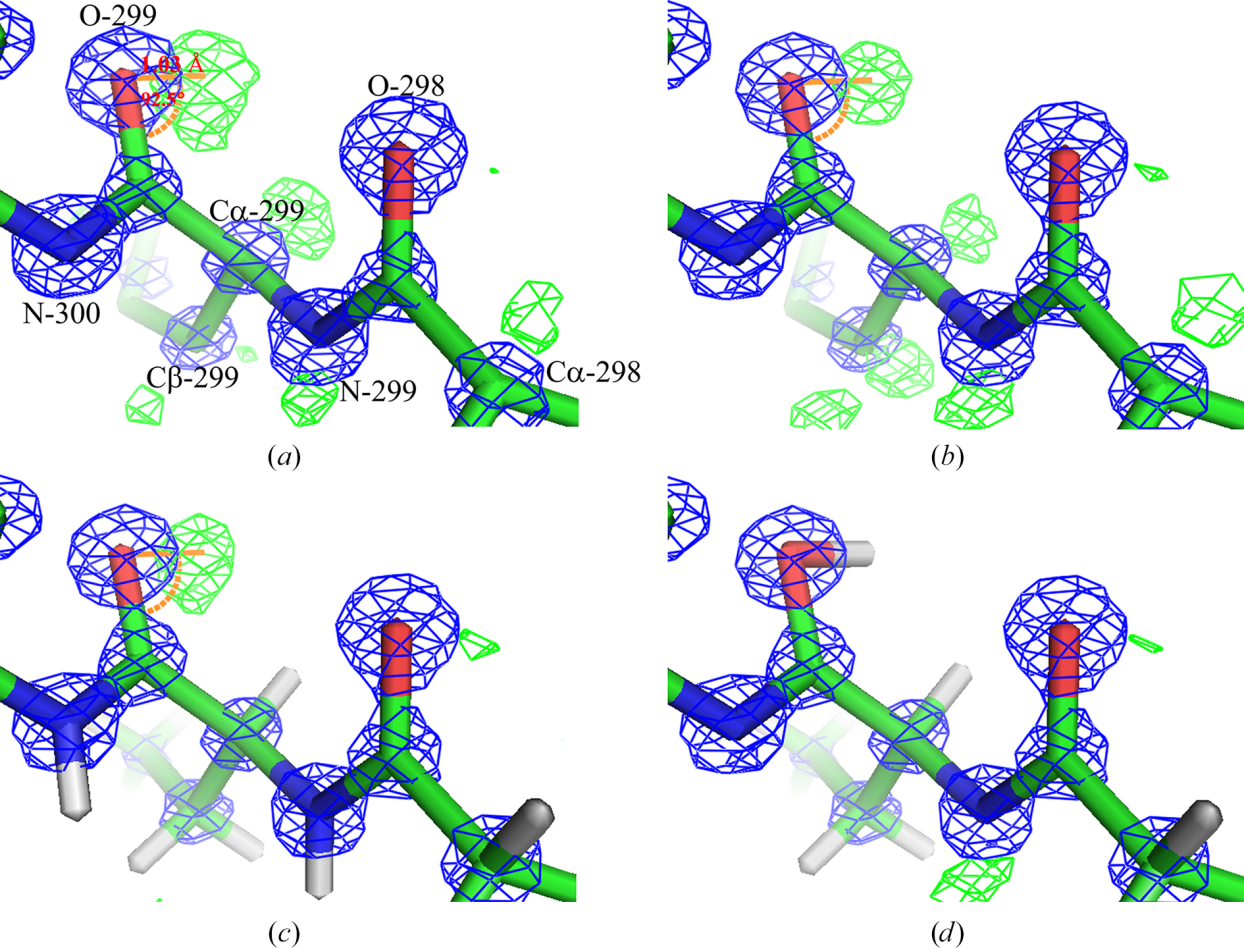
Visualization of the 2*mF*_o_ − *DF*_c_ and *mF*_o_ − *DF*_c_ maps covering residues 298–300 in β-lactamase (PDB entry 6mu9; Center for Structural Genomics of Infectious Diseases, unpublished work). The *2*mF*_o_ − *DF*_c_* map (blue mesh) represents the electron density corresponding to the refined structure, while the *mF*_o_ − *DF*_c_ map (green mesh for positive difference density) highlights regions where significant deviations are observed between the model and the experimental data. Carbon, nitrogen and oxygen atoms are shown in green, blue and red, respectively. Hydrogen atoms, where present, are depicted in white. Figures were prepared using *PyMOL* (Schrödinger). (*a*) The structure and maps correspond to the original *REFMAC*5 refinement deposited in the PDB. A difference density peak near the carbonyl oxygen atom (O-299) is observed at a distance of 1.03 Å, with a ∠C–O–peak centre angle of 92.5°. Additional difference densities are observed for hydrogen atoms at the C_α_, C_β_ and N atoms of residue 299, as well as at the carbonyl oxygen atom and C_α_ atoms of residue 298. The maps are contoured at 1.8 e Å^−3^ (2*mF*_o_ − *DF*_c_) and 0.37 e Å^−3^ (*mF*_o_ − *DF*_c_). (*b*) The structure refined in *Phenix* without hydrogen atoms shows a similar difference density pattern to that in (*a*), particularly near the carbonyl oxygen atom (O-299). The maps are contoured at 2.8 e Å^−3^ (2*mF*_o_ − *DF*_c_) and 0.37 e Å^−3^ (*mF*_o_ − *DF*_c_). (*c*) The structure refined in *Phenix* with hydrogen atoms in riding positions shows stronger difference density near the carbonyl oxygen atom of residue 299, with weaker density observed for the carbonyl oxygen atom of residue 298. This indicates an improved fit of the model to the experimental data when hydrogen atoms are included. (*d*) The structure refined in *Phenix* with hydrogen atoms in riding positions and a protonated carbonyl oxygen atom at residue 299 demonstrates further refinement accuracy. The hydrogen atom attached to the protonated carbonyl oxygen atom is well refined, with no residual difference density at this site, indicating correct placement. Additionally, the hydrogen atom from the riding position of the N atom at residue 299 was removed for validation purposes. Stronger difference density is observed near the N atom of residue 299, while weaker density is seen near the carbonyl oxygen of residue 298. Across all figures, no difference density is observed at the N atom of residue 300, validating its structural integrity. These observations highlight the importance of accurately modelling hydrogen placement and protonation states to improve structural refinement quality.

In specific cases, such as PDB entry 6mu9 (the β-lactamase penicillinase from *Bacillus megaterium*; Center for Structural Genomics of Infectious Diseases, unpublished work), a peptide bond at residue 299, located in the C-terminal helix, was observed with a dihedral angle (ω) of 179.94°. The electron density at the midpoint of the C—O bond was measured as 1.55 and that at the midpoint of the C—N bond as 2.38, suggesting a partial double-bond character in the C—N bond. A distinct difference electron density was identified at a distance of 1.03 Å from the carbonyl oxygen, with a C–O–peak centre angle of 92.5° [Fig. 4 ▸(*a*)]. Residue 299 was chosen for detailed analysis due to its strong difference electron-density peak (8σ, equivalent to 0.57 e Å^−3^) near the carbonyl oxygen, indicative of protonation, and its location within a well ordered region of the protein, minimizing potential confounding factors such as high atomic displacement parameters (ADPs) or alternate conformations. Additionally, its structural environment allowed a clear assessment of the effects of protonation on peptide-bond geometry. Interestingly, another residue, 302, in the same C-terminal helix, was also found to exhibit characteristics of a protonated carbonyl peptide.

#### 3.6.1. Refinement analysis of PDB entry 6mu9 and role of hydrogen placement and the protonated carbonyl oxygen atom

The refinement of the β-lactamase structure (PDB entry 6mu9; Center for Structural Genomics of Infectious Diseases, unpublished work) revealed significant differences in electron density and model quality depending on hydrogen placement and protonation of the carbonyl oxygen atom at residue 299.

In refinements with hydrogen atoms in riding positions, stronger difference density was observed near the carbonyl oxygen atom of residue 299, with a refined *B* factor of 6.6 Å^2^. In comparison, weaker difference density was observed near the carbonyl oxygen atom of residue 298. The inclusion of riding hydrogen atoms improved the overall model geometry, as reflected by r.m.s.d. values of 0.011 Å for bonds and 1.27° for angles.

Further refinement with a protonated carbonyl oxygen atom at residue 299 eliminated residual difference density at this site, indicating accurate placement of the hydrogen atom attached to the carbonyl oxygen. The protonated oxygen atom refined with a *B* factor of 7.9 Å^2^, confirming the stability of this model. The refinement maintained consistent r.m.s.d. values for bonds (0.012 Å) and angles (1.29°), supporting its overall accuracy.

Analysis of the 2*mF*_o_ − *DF*_c_ and *mF*_o_ − *DF*_c_ maps (contoured at 2.8 and 0.37 e Å^−3^, respectively) demonstrated no residual difference density at the N atom of residue 300 across all refinements, validating its structural integrity. Notably, in the refinement with a protonated carbonyl oxygen, the hydrogen atom from the riding position of the N-atom at residue 299 was removed for validation purposes, and a stronger difference density was observed near the N atom, further supporting the precision of the refinement.

Visualization of the maps (Fig. 4 ▸) highlights these differences. For instance, Fig. 4 ▸(*a*), corresponding to the *REFMAC*5 refinement, shows a difference density peak near the carbonyl oxygen atom (O-299) at a distance of 1.03 Å, with a ∠C–O–peak centre angle of 92.5°. Similar density is observed in refinements without hydrogen atoms [Fig. 4 ▸(*b*)]. In contrast, refinements with hydrogen atoms in riding positions [Fig. 4 ▸(*c*)] and a protonated carbonyl oxygen atom [Fig. 4 ▸(*d*)] show marked improvements, with the latter achieving no difference density near the protonated oxygen.

These results underscore the importance of accurately modelling hydrogen placement and protonation states to improve the quality and interpretability of refined structures.

Building on these findings, a dynamic refinement strategy for peptide atoms in the presence of hydrogen atoms could further enhance structural accuracy by accounting for the partial double-bond character of C=O and C=N bonds in peptide backbones. If the C=N and C=O bonds exhibit partial double-bond character, both the nitrogen and oxygen atoms may harbour hydrogen atoms simultaneously. In such cases, the occupancies of these hydrogen atoms could be refined dynamically to reflect the electronic distribution and bond character. For instance, if the C=N bond adopted a fully double-bond configuration, the occupancy of the hydrogen atom on the nitrogen atom should refine to zero, while for a fully double-bonded C=O bond the occupancy of the hydrogen on the carbonyl oxygen atom should likewise approach zero. In intermediate states where the bonds are partially double and partially single, the occupancies could refine to approximately 50% each or according to the degree of double-bond character. Implementing such a strategy could provide a more realistic representation of peptide-bond resonance and improve the quality of refined structures by accurately reflecting the underlying chemical environment. The findings from the refinement of PDB entry 6mu9 (Center for Structural Genomics of Infectious Diseases, unpublished work) not only highlight the importance of hydrogen placement and protonation states but also underscore broader implications for protein structure-refinement methodologies.

#### 3.6.2. Implications for protein structure refinement

Our findings have significant implications for the refinement of protein structures, particularly in accounting for protonation effects. The subtle impact of protonation on backbone geometry suggests that future refinement strategies should focus more on how local electronic changes influence hydrogen-bonding networks, rather than expecting large-scale geometric disruptions. Incorporating these protonation effects into structure-refinement tools could lead to more accurate models, especially in regions where hydrogen bonding plays a critical role in function, such as active sites or regions involved in protein–protein interactions.

The presence of enol forms or protonated carbonyl groups highlights the need to adapt how bond lengths and angles are restrained during refinement. Current refinement programs (for example *REFMAC*5 and *phenix.refine*) may benefit from updates that allow greater flexibility in bond angles and lengths around protonated carbonyl groups, accounting for local distortions caused by protonation. For residues showing protonation, less stringent restraints could enable more accurate modelling of the enol form and its associated bond angles. This flexibility would better capture the dynamic and varied nature of these structural modifications, ultimately improving the fidelity of protein models in regions where protonation plays a structural or functional role.

#### 3.6.3. Protonation effects in β-strands

Protonation of the carbonyl oxygen atom in β-strands can have a significant impact on hydrogen bonding, which is essential for stabilizing β-sheets. When the carbonyl oxygen atom is protonated, it reduces its ability to serve as a hydrogen-bond acceptor, as its lone-pair availability is diminished. This effect leads to elongated and weakened hydrogen bonds, consistent with previous findings on oxoacid protonation (Perrin & Nielson, 1997 ▸; Gilli *et al.*, 1989 ▸). Such weakening can lead to a reduction in inter-strand hydrogen-bond strength, potentially compromising the stability of the β-sheet.

Protonation may also introduce minor structural distortions, such as increased flexibility in the peptide backbone, making it more challenging for strands to align optimally. Computational studies have demonstrated that protonation alters electron delocalization within the peptide bond, promoting enol-like character, which weakens conventional hydrogen bonding (Szostak *et al.*, 2015 ▸; Scheiner & Wang, 1993 ▸). This can result in looser hydrogen-bonding networks, reducing the overall structural integrity of β-sheet-rich regions, which are typically rigid and highly ordered, as seen in amyloid structures or fibrous proteins.

To further investigate the environmental context of protonated carbonyl oxygens in β-strands, we analysed their solvent accessibility and interactions with water molecules. Out of 332 protonated carbonyl oxygen atoms identified in β-strands, 147 (44%) were solvent-exposed, while 185 (56%) were buried. Among the solvent-exposed residues, 87% (128 out of 147) formed hydrogen bonds with water molecules, indicating a potential role of hydration in stabilizing protonated states. Even within buried regions, structured water molecules were found in contact with 49% (91 out of 185) of protonated carbonyl oxygens, suggesting that hydration pockets may facilitate protonation even in shielded environments.

Structural studies of protonated carbonyl oxygen atoms indicate that hydrogen bonds involving C–O–H groups tend to be longer and weaker compared with conventional C=O acceptors, supporting the idea that protonation may locally increase backbone flexibility (Limbach *et al.*, 2009 ▸). The presence of protonated carbonyl oxygen atoms in both solvent-exposed and buried regions highlight that protonation is not solely dictated by solvent accessibility but is also influenced by local hydrogen-bonding networks and structured water molecules. This suggests that protonated β-strands may be more adaptable, with increased hydrogen-bond fluctuations, which could play a functional role in certain proteins by modulating local stability and flexibility.

#### 3.6.4. Protonation effects in α-helices

In helices, protonation of the carbonyl oxygen atom introduces localized disruptions that can significantly impact the regular hydrogen-bonding pattern essential for helical stability. Typically, each carbonyl oxygen atom in a helix forms a hydrogen bond with the amide hydrogen four residues down the chain, creating a compact and stable structure. However, protonation neutralizes the partial negative charge of the carbonyl oxygen, weakening the overall helical dipole. Similar reductions in dipole moments due to protonation effects have been observed in oxoacid systems, where hydrogen-bonding strength is directly impacted by electronic redistribution (Limbach *et al.*, 2009 ▸; Hibbert & Emsley, 1990 ▸).

Protonation of a carbonyl oxygen atom within a helix may weaken or distort these hydrogen bonds, potentially leading to local unwinding or increased flexibility in the affected region. This effect can be particularly impactful near the N- or C-terminal ends of helices, where flexibility is naturally higher.

In the core of the helix, however, protonation may disrupt the continuous hydrogen-bonding network, which could destabilize the helical turn and affect the overall stability of the helix, especially in regions critical for helix–helix or helix–sheet packing within the tertiary structure of the protein. Computational studies suggest that protonation may alter electron-density distributions, thereby weakening hydrogen-bonding patterns and subtly influencing protein stability and folding behaviour (Szostak *et al.*, 2015 ▸).

To further investigate the structural environment of protonated carbonyl oxygens in α-helices, we classified their solvent accessibility and interactions with water molecules. Out of 527 protonated carbonyl oxygens identified in helices, 311 (59%) were solvent-exposed, while 216 (41%) were buried. Among solvent-exposed residues, 82% (255 out of 311) formed hydrogen bonds with water molecules, suggesting that hydration plays a role in stabilizing protonation. Even among buried residues, 42% (91 out of 216) exhibited interactions with structured water molecules, indicating that local hydration pockets could facilitate protonation even in the helical core. These findings suggest that hydration and solvent exposure may influence protonation patterns, but protonation is not exclusively limited to solvent-accessible regions.

In addition to its structural effects, protonation also influences the helix dipole moment. In a helical structure, the alignment of peptide dipoles from each C=O and N—H bond in the backbone creates a cumulative helix dipole moment, with a partial positive charge at the N-terminus and a partial negative charge at the C-terminus. This cumulative dipole is due in part to the partial negative charge of each carbonyl group pointing toward the C-terminus of the helix.

Protonation of a carbonyl oxygen atom disrupts the regular distribution of dipoles along the helix axis and reduces the contribution of that carbonyl group to the overall dipole moment. If multiple carbonyl groups are protonated, the reduction in dipole moment could be more pronounced, potentially weakening the macrodipole of the helix. This change could impact the ability of the helix to interact with other charged molecules or regions within the protein.

The helical dipole plays a role in stabilizing interactions with ligands, ions and other protein regions, particularly those with opposite charges (Hol, 1985 ▸). Changes in the dipole moment due to protonation may reduce binding affinity in cases where the helix dipole contributes to electrostatic interactions, such as in active sites or binding interfaces. Additionally, the presence of protonated carbonyl oxygens in both solvent-exposed and buried regions indicates that protonation is influenced by a combination of solvent accessibility, local hydration and intra-helical hydrogen-bonding networks rather than a single factor. This alteration could also influence folding and stability, as dipole–dipole interactions within the protein are often critical for maintaining tertiary and quaternary structure.

### 3.7. Robustness testing and validation across refinement and resolution criteria

To assess the robustness of the findings and minimize potential biases introduced by refinement parameters, additional analyses were conducted using more stringent *R*-factor and refinement thresholds. Subsets of 748 structures (*R* ≤ 0.15) and 700 structures (*R*_free_ ≤ 0.175) were examined to evaluate the impact of stricter refinement criteria. Additionally, 685 structures refined with anisotropic atomic displacement parameter (ADP) models were analysed to ensure a more accurate treatment of atomic motion. A smaller subset of 30 structures (*R* ≤ 0.10) was also considered; however, its limited sample size was deemed to be insufficient for statistically reliable conclusions.

Beyond these refinements, a prior independent analysis using only structures with resolution ≤1.0 Å was performed, yielding results fully consistent with the present study. Across all data-set variations, key geometric trends, electron-density distributions and hydrogen-bonding properties remained unchanged, indicating that the conclusions are not artefacts of data-set selection but rather reflect intrinsic structural features of peptide bonds.

To further validate these findings, a focused analysis of the 685 anisotropic ADP-refined structures was conducted independently. The observed trends, including bond lengths, bond angles, dihedral angles, electron-density ratios and hydrogen-bonding characteristics, remained consistent across this rigorously curated data set. Similarly, verification using the independent ≤1.0 Å resolution data set produced comparable results. These findings confirm that the differences in peptide-bond geometry and electron-density distributions are intrinsic structural properties rather than artefacts of refinement protocols.

The strong agreement across multiple high-resolution data sets reinforces the robustness and generalizability of the conclusions of this study.

## 4. Conclusions

In this study, 1024 high-resolution, nonredundant protein structures were analysed, and 579 structures (∼56.5%) were identified to contain a total of 1589 peptide bonds with potentially protonated carbonyl oxygen atoms. Protonation was inferred from the presence of significant difference density peaks near the carbonyl oxygen atom and was further verified through analysis of peptide-bond dihedral angles. Despite protonation, the peptide-bond geometry, including bond lengths and angles, remained largely consistent with the typical values for partial double bonds, particularly around 1.33 Å for the C—N bond. Minimal differences in bond lengths were observed between helices and strands, while slight variations in bond angles, particularly in strands, suggested a more relaxed geometry that may contribute to their adaptable structural roles in proteins.

Our findings on dihedral angle distributions underscore distinct conformational preferences in helices and strands: helices exhibit a stable, tightly clustered distribution around 180°, whereas strands span a broader range, highlighting structural adaptability. The protonation of carbonyl oxygens did not markedly alter the backbone conformation, as confirmed through Ramachandran analysis. However, protonation may still influence hydrogen bonding and local flexibility, especially in β-strands, where structural flexibility is more pronounced. Such subtle electronic effects on backbone geometry have important implications for protein dynamics and folding, potentially impacting protein regions essential for functional adaptability under physiological conditions.

Beyond structural stability, the findings provide valuable insights into the role of protonation in secondary-structure stability, suggesting that protonation may subtly influence local folding dynamics and intermolecular interactions. Importantly, this study highlights how protonation of carbonyl oxygens in α-helices could alter hydrogen-bonding patterns and affect the helical dipole moment, potentially impacting the electrostatic interactions of the helix with other charged biomolecules.

Our findings emphasize the necessity of refining structural models in a way that captures true geometric variability while maintaining appropriate restraint weighting. Future studies could explore re-refinements across a larger data set to systematically evaluate the extent to which bond-length and angle deviations persist across diverse protein structures. Additionally, alternative refinement strategies, such as dynamic restraint weighting or quantum crystallography approaches, may further improve the accuracy of peptide-bond representations in structure-refinement protocols.

Future studies could explore *PDB-REDO*-based comparisons to assess the influence of standardized re-refinement protocols on peptide-bond geometries (van Beusekom *et al.*, 2018 ▸). While our data set consists of high-resolution structures where refinement effects are expected to be minimal, *PDB-REDO* refinements may provide additional validation, particularly for evaluating restraint biases in bond-length and angle constraints. Given the consistency observed across different refinement criteria, we anticipate that *PDB-REDO* would reinforce rather than alter our findings. However, such an analysis could be valuable for refining restraint models in crystallographic refinement software, improving structural accuracy in future studies.

In the context of protein structure refinement, these insights emphasize the need for refinement programs to account for localized electronic changes without rigid geometric constraints. Programs such as *REFMAC*5 and *phenix.refine* could benefit from incorporating flexible bond-length and angle restraints around protonated carbonyl groups to better represent the dynamic nature of protein structures. Integrating these protonation effects into refinement algorithms may yield models that more accurately capture the behaviour of protonated or enol-like peptide bonds, particularly in active sites, binding regions or protein–protein interfaces where precise structural representation is essential.

This work not only advances our understanding of protonation effects on peptide-bond geometry but also sets the foundation for future studies to explore the interplay between peptide-bond chemistry and protein function. Such insights could contribute to novel approaches in protein engineering and therapeutic design, leveraging our enhanced knowledge of protein stability and adaptability in response to protonation.

While this study relies on IAM-based electron-density maps, future investigations could benefit from alternative refinement approaches that better capture deformation density. Methods such as multipolar refinement (Hansen & Coppens, 1978 ▸) and quantum crystallography (Jayatilaka & Dittrich, 2008 ▸) provide a more accurate representation of electron-density distribution and could offer additional validation for the trends observed here. Incorporating these advanced methodologies in peptide-bond studies may further elucidate the electronic factors contributing to secondary-structure stability and bond delocalization.

## Supplementary Material

. DOI: 10.1107/S2052252525002106/jt5080sup1.pdf
Supplementary Table S1 and Figure S1

## Figures and Tables

**Table 1:** Bond lengths and bond angles

	Single bond	Double bond	Peptide bond
Bond lengths (Å)			
C—N	1.47	1.27	1.32
C—O	1.43	1.21	1.24
Bond angles (°)			
∠OCN	—	—	123.2
∠C_α_CN	—	—	115.6
∠CNC_α_	—	—	121.9

**Table 2:** Refinement statistics for PDB entry 6mu9 and its variants with hydrogen atoms in riding positions, including the protonated carbonyl oxygen atom at residue 299

PDB code	6mu9 (as reported in the PDB)	6mu9 refined in this work	6mu9 refined with hydrogen atoms in riding positions	6mu9 refined with protonated carbonyl oxygen atom at residue 299
Refinement program	*REFMAC*5	*Phenix*	*Phenix*	*Phenix*
Resolution range (Å)	20–0.97	20–0.97	20–0.97	20–0.97
*R* factor/*R*_free_ (%)	10.1/11.1	10.83/12.79	9.39/11.05	9.40/11.05
No. of hydrogen atoms	0	0	2240†	2239†
No. of water molecules	430	430	430	430
R.m.s.d., bond lengths (Å)	0.009	0.008	0.011	0.012
R.m.s.d., angles (°)	1.49	1.11	1.27	1.29
*B* factors of peptide atoms (Å^2^)				
C atom of residue 299	5.6	6.2	5.9	6.1
O atom of residue 299	6.3	6.7	6.6	6.6
N atom of residue 300	5.5	6.0	5.8	5.8
H attached to N atom of residue 300	—	—	5.3	—
H attached to O atom of residue 299	—	—	—	7.9

† Fig. 4 ▸(*d*) deliberately omits the hydrogen atom on N-299 (resulting in 2239 H atoms in refinement number 4) as part of the validation process to highlight the difference density for hydrogen.
