# Supplementary material for: Peptide bonds revisited
Source: IUCrJ. 2025 Mar 31;12(Pt 3):307–21. doi: 10.1107/S2052252525002106 (PMC12044857; doi:10.1107/S2052252525002106)
Supplement: Supplementary file 1 [file m-12-00307-sup1.pdf]

IUCrJ

Volume 12 (2025)

Supporting information for article:

Peptide bonds revisited

Santosh Panjikar and Manfred S. Weiss

Table S1 List of PDB codes and corresponding chain IDs used in this study.

The table presents the 1,024 unique PDB entries, each associated with a chain ID.

1EJGA 5NW3A 1UCSA 2VB1A 7VOSA 1US0A 6E6OA 6S2MA 1R6JA 4REKA 4I8HA
2WFIA 2OV0A 2B97A 8C5NA 3X34A 7KR0A 6L27A 5YCEA 7A5MA 5GV8A 6ZM8A
1X6ZA 4UA6A 5TDAA 3UI4A 5AL6A 5NFMA 1W0NA 5HB7A 2H5CA 2VXNA 1NWZA
8C3XA 7AVKA 2JFRA 2O9SA 4Y9WA 6TGUA 1P9GA 7BNHA 2O7AA 2YKZA 4EICA
4AYOA 6Q00A 3O4PA 6ETLA 1MC2A 1X8QA 7BBXA 7AOTA 3QPAA 5EMBA 2FMAA
5XVTA 2F01A 1G6XA 3WDNA 1MUWA 6ZPAA 2DDXA 7R25A 8EREA 4HS1A 5L87A
5O99A 1GWEA 3ZOJA 3FILA 4F1VA 5LP9A 1I1WA 4U9HL 4O6UA 4UYRA 3IP0A
8AVUA 4WEEA 4Y9VA 1IX9A 6KLZA 7OUZA 4NPDA 1VYRA 5X9LA 7PSYA 3VORA
3G21A 5GJIA 3ZR8X 4EA9A 6XVMA 2XU3A 6LK1A 5A71A 5IG6A 3G46A 5BR4A
3X0IA 7P1QA 8CR4A 5MK9A 4HNOA 1IQZA 6EQEA 3EA6A 4G78A 6CNWA 1L9LA
3WGXA 6ZSYA 1VBWA 3QL9A 3M5QA 4R2XA 2BT9A 3QR7A 2UU8A 2GUDA 4MZCA
1BXOA 6TWTA 2XOMA 2XJPA 5U3AA 7A3HA 1MJ5A 4KQPA 5O2XA 3S6EA 4XDXA
3DK9A 1ZZKA 3VLAA 4G9SA 4E3YA 5EWOA 3DHAA 6J93A 3NEDA 7DC4A 4EGUA
1NKIA 3F1LA 4GA2A 6IIPA 1UFYA 7Q0OA 3ZZPA 1K5CA 3PUCA 5DP2A 3AGNA
5JUGA 2XODA 2E4TA 3KFFA 6JPTA 3E4GA 4R5RA 7Z8OA 1AHOA 1U2HA 2XFRA
1BYIA 4ACJA 6X7JA 7RWGA 5DZEA 5TIFA 3EO6A 1ZUUA 4Q4GX 3VIIA 1M1QA
4OO4A 7L71A 8B97A 4PNOA 3GOEA 1XMKA 4MTUA 6YP6A 8JEAA 6FJNA 3AKSA
6RYGA 6Q7DA 3V1AA 2V8TA 4HGUA 3D1PA 5SY4A 3JU4A 3FSAA 3PSMA 7YZ7A
4EKFA 4WPKA 4A7UA 2PNEA 1UNQA 3JUDA 5SBQA 4IAUA 1MWQA 3Q46A 5CKLA
2FWHA 5O45A 7KOMA 6RK0A 4AQOA 6RVUA 6G1IA 4GZNC 8A55A 6U66A 5II8A
4NDSA 4X5PA 5JDKA 6YK4A 2GKGA 7ADRB 1EB6A 3AJ4A 1O7JA 3FYMA 4N1IA
4BJ0A 2CNQA 1LNIA 6IGGA 4ZGFA 5WS7A 2R31A 2GGCA 1TT8A 1Q6ZA 2RBKA
4YMYA 2JHFA 3NOQA 4GNRA 5CTMA 2A6ZA 4CNNA 1OD3A 3RWNA 5SV5A 1MN8A
6GZ8A 3L8WA 2VHAA 4AXOA 2X46A 6FVIA 2P5KA 5D66A 5DGJA 5JIGA 2CE2X
3RQ9A 8E77A 3WH1A 7K9CA 1OAIA 8AXJA 4JP6A 6I3BA 1C7KA 6UKFX 1ZK4A
3A4RA 3SOJA 4TXRA 3JYOA 6L8GB 3BWHA 3A02A 2V1MA 3CCDA 7SNEA 7B5WA
3ZUCA 4YPOA 3TEUA 6R33A 3SK2A 7JJAA 4BPFA 6Z5YA 8BJ8A 3NBCA 5JH8A
6Y4EA 6QAZA 5SSXA 1CXQA 5MDUA 6I03A 4YSIA 7T5UA 5AOTA 3NVSA 5YDEA
7RM7A 1KQPA 4UHCA 5M17A 5Y0MA 6EF7A 4UDXX 3B64A 7URPA 3ROFA 1Y93A
4XPXA 1XQOA 8G53A 5D5YA 5ONKA 3VZ9B 6DCMA 3QZRA 6N9HA 3A72A 2OB3A
7QTBA 4RV5A 5TNVA 6FM7A 5IMAA 2ANXA 7T3EA 3I94A 5AE0A 2HEUA 7X44A
5P9VA 6MM2A 2R16A 2BOGX 3CUZA 1I2TA 4B9GA 1D5TA 7BGGA 6BEVA 8AYVA
4GJZA 3C70A 6RY0A 5WGIA 8Q9XA 1PSRA 6F9OA 1YQSA 4CO8A 6TVEP 2OIZA
7E2SA 5NWPA 7D8DA 2V3IA 6NNRA 1FSGA 4X2RA 5FI3A 3LO8A 2C71A 7MPDA
3GMXA 4MNCA 2VZPA 5B8DA 4PLZA 3IQUA 6SIDA 2X5YA 4W7LA 3R2QA 4B5OA
5FAFA 1LQTA 2D5MA 5CTVA 5QI0A 3WMVA 3VUPA 4ANNA 6WQYA 3PB6X 6S1HA
4W7WA 1EUWA 1JCLA 2VZCA 6C1XA 6TCCA 6ELVA 3R87A 7ESKA 2AXWA 7LKLB
2HINA 3G5SA 4CO3A 7Z09A 1KJQA 6R4ZA 7ET5A 6H40A 7TFMA 4D8BA 6B8FA
4XOTA 3TC8A 8G1LA 4FK9A 3ULJA 4UZGA 4KGDA 4E9SA 3W42A 3MCWA 1N40A
3NYCA 6YOOA 7BIUA 8GKOA 2DKOA 2R0XA 8CMPA 6JK4A 7CKAA 7BLKA 5HUBA
8PHYA 8GQQA 1SFSA 4Q68A 6GX2A 5TFQA 8BBPA 3CIJA 3VGIA 2O90A 4QRNA
5W0GA 5OLRA 6V67A 8AIFA 6S5WA 6WFNA 7CX5A 4Q2LA 4K12B 1JBEA 2CI1A
6TM3A 1W23A 1W66A 4M51A 6P2LA 8UWVA 2XW6A 3WA2X 2YHGA 6SE1A 4L57A
5OJ5A 7R5IA 6EKZA 6FIHA 5LUNA 7O4PA 4BPSA 6GY5A 4G9EA 2CARA 1NKDA
6THOA 6ZEGA 6FIYA 7AVPA 4WUIA 4ZO2A 8D0PA 1QLWA 4GS3A 5Y9ZA 3ZY7A
5J1NA 3T7LA 4YTKA 6FTFB 5OHQA 4KEFA 4RXVA 1C5EA 1ZL0A 3RPEA 4CJ0A
5Z3EA 6T85A 3AYJA 8B2EA 5EL9A 2Z72A 4WWFA 3LWXA 7ZTFA 4A9VA 6RRVA
6RZ0A 2AIBA 4A29A 3RO3A 2V9VA 2OSXA 6R1DA 4HE6A 8SA9A 4ATEA 2NWFA
5JBXA 3QZBA 8BBUA 7YUGA 7LV6B 2C2UA 3E8MA 1GMXA 2BHUA 4QLPB 3KWEA
4LF0A 1SBYA 1Z2UA 4H7WA 6G7NA 4JZ5A 6MU9A 4CVRA 3P4HA 7O91A 4MAKA
4J8CA 2ABSA 1LS1A 2W39A 5T39A 2NXVA 4M91A 4MIJA 3RZNA 8EHEB 8C86A
2VFRA 5OD4A 6W2GA 3V0DA 1BKRA 6E1ZA 4XUWA 6EXXA 1UZ3A 2FBAA 4UE8A
2V8FA 3CT6A 1RG8A 6DYFA 3NZNA 3SU6A 3BVXA 4CD5A 4PF3A 2I5VO 4ETNA
4B1MA 4ES1A 5GGBA 5LJPA 7Q6AA 4CNGA 6KP5A 3MVSA 4BT7A 4QA8A 3LQBA
7S7WA 5D7WA 2RHFA 3MREA 4NI6A 7F9JA 6YV5A 7W0QA 6T02A 8TNMA 7TBRA
2II2A 3TG2A 5IWHA 6JQBA 5VNYA 3U97A 6H10A 6DQHA 7Z65A 4MF5A 3ORUA

6QTSA 6TL7A 7QCPA 3A8GB 5WSFA 5OUOA 5W0HA 3CI3A 4YAPA 6OSHL 3G5TA
5N6FA 1SAUA 1K7CA 2YLNA 6PZDA 4HCJA 6DTVA 6TTNA 5MX9A 5EMIA 3U01A
6RG2A 1TUKA 4CK4A 6E7EA 8ONBA 2GZVA 5UFYA 2WJ5A 4YECB 6GHTA 6SYVA
1RA0A 6LUJA 2EABA 4KNKA 4ZURA 3JQ0A 5GTQA 5J4LA 6HPHA 6ZC2A 1U07A
4BPZA 6N4LA 6QDIA 7TOIA 3D9XA 6SWIA 6YHMA 7DQTA 7P5BA 6Q9LA 7LF3A
6AO9A 4JCCA 4RVQA 4WFOA 1P6OA 5AOZA 7UX7A 5UM2A 5UE1A 8G0NA 8E18A
4BK7A 4CQHA 6ELMA 4W8HA 6W1GA 7T26A 4QPWA 4INWA 5KLAA 6JV0A 7A73A
8JA1A 3S2RA 6ZNVA 5KARA 6HFQA 5UQZA 2ZK9X 7CN7C 4JXRA 4YZ0A 3DLCA
4UU3A 4WN5A 2OLNA 4N03A 8C8FA 5NQOA 6NP3A 6H8OA 4H3UA 1GWMA 6T9QA
7W2PA 3ZZOA 6CWMA 3W06A 2NSZA 4JN7A 3DNJA 4EZIA 4KU0A 7PQKA 2V7FA
4DT5A 4ZVFA 6K7CA 4JK8A 8SBNA 3M73A 7W50A 1ODMA 7LDQA 4U98A 6GEHA
2ZPTX 2R01A 1XMTA 7S7JA 2CIWA 1I4UA 6UAQA 3R72A 3S0AA 2IC6A 7M10A
5G38A 6NLQA 7MQQA 4BEUA 4HROA 3T3LA 5BTYA 3H4TA 4O06A 5K34A 7SOQA
5JXMA 3BFOA 2DKJA 8IN3A 5U5OA 7XTNA 2FHZA 6CPBA 5AIGA 5LS7B 4AL0A
5Z0DA 6C4QA 1H4XA 6E1FA 3C6AA 7P2MA 5O0SA 1X9IA 7ZPGA 6RVPA 6L7QA
7BNBA 5FEWA 5A6MA 7LWEA 4UE0A 1H97A 5FYPA 4RJZA 3X0TA 8H0TA 6B1KA
6SJ3A 6FUCA 6ENNA 4EMNA 5YKZA 7Q5VA 6PZLA 1WKQA 2OFZA 4RU3A 5W8QA
5O15A 4NNOA 4BFOA 5TZMA 5F6RA 6A9SA 6SSDA 3WDCA 1KQ6A 5ZHZA 7BXDA
4NLMA 6MRRA 6T6HA 5EQ0A 4JHTA 7XMHA 4TPNA 8IUBA 5V6JA 7GEFA 5Q22A
5G5CA 1N3LA 5K91A 5UQ6A 8B2FA 5DICA 5EQ7A 3A5FA 4YEPA 5ZB0A 4FS7A
3S6FA 8BFTA 1UWKA 4RLZA 5LW3A 3UJCA 1GU2A 7TVCA 5J41A 6DGAA 2XIOA
5JQNA 4LLDB 6P7ZA 6S95A 5SZCA 6IUXA 6ETCX 4X9XA 5UUKA 1SENA 6HERA
8CI6A 7NZAA 4OD6A 2BK9A 5E9PA 1H12A 6SAOA 6NIBA 2NLRA 5JRYA 6I6MA
2V3GA 3IR4A 3HYNA 4PQ9A 2FKKA 1VR7A 6GD6A 4RGDA 7XIHA 4IGIA 2IAYA
5AGDA 4YI8A 6SLLA 5MFAA 5HHJA 7AT0A 6BO0A 3BWZA 1C0PA 1M15A 8FUXA
4ZJUA 5BY5A 5L4LA 3B4UA 3M0ZA 1XDNA 4D0QA 3OHEA 3VWNX 8A7CA 2H1VA
3H5JA 4L8AA 7QOWA 3BONA 3GNEA 6UFEA 8B6EA 6XFJA 4NOGA 3VQJA 2ICCA
3IPJA 2BLNA 5CWGA 3SQZA 4UASA 1Z0WA 2GXQA 8AQKB 3EDOA 6SPOA 5OXZA
4EBGA 6PQKB 3OG2A 4WWHA 2X5XA 3FRHA 4N6KA 8C4PA 5WQJA 4NYHA 6D9NA
5K2LA 7EHUA 5I39A 2FCLA 6CBUA 4P40A 6A56A 8BRSB 5FSVA 2QF4A 6I9AA
6SU5A 4NYQA 5V2OA 4FR9A 7ZOBA 4LUPA 3OXPA 2IJ2A 3A9BA 4KV7A 6T0YA
6KFSA 5GJHA 2PGNA 2ZNRA 7JGUA 6FU9B 6SWTB 4UQXA 2OMLA 1W6SA 7EZIA
7QOEA 2DLBA 6DT3A 2R2ZA 2BMOA 2ZEXA 7VZPA 5TOQA 6Q0MA 6QXRA 3BUUA
4GWBA 5IGIA 6HBBA 3FWKA 6YSBA 1NZ0A 4IIYA 8DAJA 4TTWA 1TU9A 4O0AA
4BGCA 1VK1A 6UOFA 5MR1A 1UAIA 5M1NA 2W5QA 3A6RA 1X6IA 4GMUA 1QW9A
7BBVA 4RFUA 7EYLA 2A26A 8F3AA 3N17A 3B12A 3D06A 2VK2A 2ZFDB 2H8EA
1Z2NX 2NRRA 3DQYA 3R9FA 3F0DA 6QXUA 2CG7A 2VY8A 1I24A 1WN2A 1O7IA
3G36A

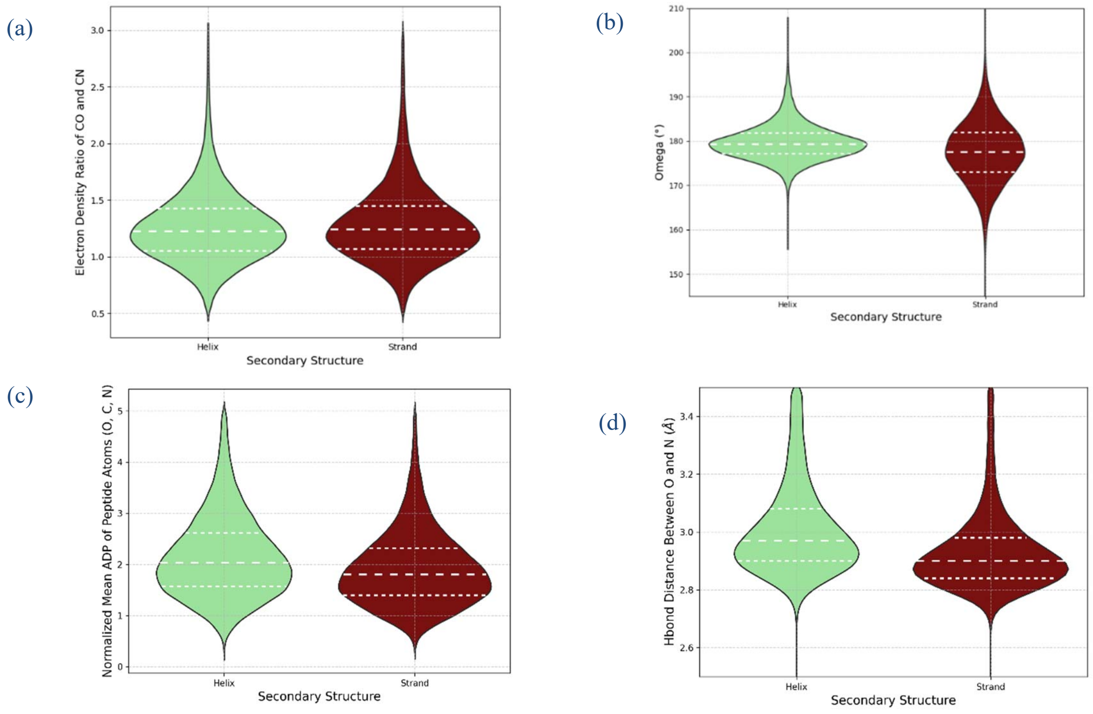

Figure S1 Violin plots illustrating structural and electronic properties of peptide bonds in α-helices and β-strands. These violin plots provide a complementary visualization to Figure 3 in the main manuscript, which presents the same parameters using KDE plots. The violin plots display the full distribution of data while also showing quartile divisions (dotted white lines), allowing for a clearer comparison of spread and density between α-helices and β-strands. (a) Electron density ratio of CO and CN bonds: The distribution of the electron density ratio (D_{ratio}) between the carbonyl (C=O) and amide (C-N) bonds, highlighting differences in electronic delocalization between helices and strands. (b) Peptide bond dihedral angle (Ω): The distribution of the peptide bond dihedral angle (ω), showing how secondary structure influences backbone planarity and flexibility. (c) Normalized mean ADP of peptide atoms (O, C, N): The distribution of normalized atomic displacement parameters (ADP) for peptide backbone atoms (O, C, and N), reflecting differences in structural flexibility between helices and strands. (d) Hydrogen bond distance between O and N atoms: The distribution of hydrogen bond lengths between backbone oxygen (O) and nitrogen (N) atoms, demonstrating differences in hydrogen bonding patterns in helices and strands. Each violin plot compares α-helices (light green) and β-strands (dark red), with dotted white lines representing quartiles.
